# Artificial Intelligence use cases adopted by people and their impact on achieving sustainable development goals: a systematic review

**DOI:** 10.12688/openreseurope.20023.1

**Published:** 2025-04-28

**Authors:** Vijay Palliyil, Meng Cai, Hiba Karam, Lalita Phatthanachaisuksiri, Niklas Suhre, Eva Kaßens-Noor

**Affiliations:** 1 Department of Civil and Environmental Engineering, Institute of Transportation Planning and Traffic Engineering, Technical University of Darmstadt, Darmstadt, Hessen, 64287, Germany; 2 School of Planning, Design and Construction, Michigan State University, East Lansing, Michigan, 48824, USA

**Keywords:** Artificial Intelligence, Sustainable Development Goals, People

## Abstract

Individuals are increasingly integrating Artificial Intelligence (AI) into their lives, adopting various use cases in healthcare, education, urban mobility, and more. AI has the potential to enhance efficiency, well-being, and societal progress, but it also has negative potential associated with ethical challenges, privacy concerns, and social inequality. A significant research gap remains in understanding the impacts of AI use cases adopted by people on SDG achievement. This study addresses that gap through a systematic analysis of whether AI adoption by people supports or hinders progress toward the SDGs. Using the PRISMA framework, we conducted a systematic review of 131 studies. The results show that the overall impact of AI use cases adopted by individuals on the SDGs is moderately positive. These use cases significantly contribute to areas such as healthcare, innovation, and sustainable urban development, yet their effects remain complex and context dependent. While individually adopted AI fosters efficiency and well-being in many domains, concerns about job displacement, biased decision-making, and misinformation highlight the need for responsible deployment. The study emphasizes the importance of ethical AI governance, equitable access, and AI literacy to ensure its positive contribution to sustainable development. Future research should not only empirically evaluate the real-world impacts of AI applications adopted by people from a sustainability perspective but also explore and develop strategies to mitigate negative impacts on progress toward the SDGs while maximizing their positive contributions. This research contributes to the evolving discourse on AI adoption by people and its implications for sustainable development.

## Introduction

Artificial Intelligence (AI) applications have become increasingly prevalent, with people adopting a wide range of use cases that enhance various aspects of daily life, from personalized healthcare ( Al-Besher & Kumar, 2022; König, 2023; Yigitcanlar *et al.*, 2024) and education ( Choung *et al.*, 2023; Si, 2022; Yeh *et al.*, 2021) to smart home technologies ( Choi & Drumwright, 2021; Das *et al.*, 2023; Maninger & Shank, 2022) and urban mobility solutions ( Gesk & Leyer, 2022; Kleizen *et al.*, 2023; Oleksy *et al.*, 2023). AI use cases have the potential to positively impact people’s lives by improving quality of life, enhancing time and cost efficiency, and contributing to broader societal advancements, thereby supporting the achievement of the United Nations' Sustainable Development Goals (SDGs) ( Ametepey *et al.*, 2024; Goralski & Tan, 2020; Gosselink *et al.*, 2024). However, alongside these positive potentials, the adoption of AI also poses challenges, including ethical dilemmas, data privacy concerns, and risks of reinforcing social inequalities ( Ingram, 2021; Lainjo, 2024; Sartori & Theodorou, 2022; Si, 2022; Vinuesa *et al.*, 2020).

AI use cases span a wide range of applications, bringing diverse impacts that can be both positive and negative ( Kinder *et al.*, 2023; Lainjo, 2024; Ranga, 2024; Sætra, 2021; Si, 2022). On the positive side, AI has enhanced learning experiences through personalized education platforms ( Das *et al.*, 2023; Murthy *et al.*, 2024), improved healthcare through accurate diagnostics ( Robinson, 2020; Yeh *et al.*, 2021) and predictive modelling ( Ozmen Garibay *et al.*, 2023), and optimized urban living with smarter traffic ( Al-Besher & Kumar, 2022; Moravec *et al.*, 2024) and resource management systems ( Yigitcanlar *et al.*, 2023). On the flip side, some use cases have raised concerns, such as job displacement caused by automation ( Albarrán Lozano *et al.*, 2021; Liang & Lee, 2017), the amplification of biases in AI-driven decision-making ( Langer *et al.*, 2023; Sartori & Theodorou, 2022), and significant environmental costs due to the energy demands of large-scale AI operations ( Spelda & Stritecky, 2020). These varied outcomes underscore the importance of examining each AI use case individually to understand its unique implications and to implement it responsibly.

Most studies about AI and people focus on its sector-specific applications, while others explore people's perceptions, attitudes, acceptance of AI, or its impact on various aspects of human life. However, there is a significant gap in understanding the diverse use cases of AI adopted at the individual level and their impacts on the Sustainable Development Goals (SDGs). Some studies have evaluated the overall impact of AI on the Sustainable Development Goals (SDGs), considering AI as a monolithic entity ( Ametepey *et al.*, 2024; Vinuesa *et al.*, 2020). Others have examined selected case studies of AI applications, demonstrating how these specific applications of AI impact the SDGs ( Goralski & Tan, 2020). This paper addresses this gap by systematically analysing academic literature, cataloguing AI use cases adopted by people, and evaluating their diverse impacts on achieving the SDGs. The individual use cases of AI are highly diverse, leading to varying impacts on the Sustainable Development Goals (SDGs) in terms of which goals they affect, whether the effects are positive or negative, and the extent of their influence. Hence, a systematic analysis, like the one undertaken in this study, is crucial to understanding their potential to drive progress or introduce challenges.

## Method

We conducted a systematic review using the Preferred Reporting Items for Systematic Reviews and Meta-Analyses (PRISMA) protocol. The PRISMA protocol ensures transparency and consistency, making it easy for the reader to understand the selection process and improving the quality of the systematic review ( Knobloch *et al.*, 2011), and The PRISMA procedures are divided into four phases: (a) identification, (b) screening, (c) eligibility, and (d) inclusion ( Moher *et al.*, 2009). 

(a) In the identification phase, we used Web of Science (WOS) and Science Direct (SD) to identify scientific studies. WOS, owned by Clarivate Analytics, is a large multidisciplinary citation database spanning all disciplines and SD, provided by Elsevier, is the world's leading database of peer-reviewed, full-text scientific, technical, and health literature. We searched both databases using titles and abstracts, with the search terms (("people" OR "citizens" OR "residents") AND ("artificial intelligence" OR "AI")).

Furthermore, we defined the scope of the studies of interest in alignment with our research aim, following Moher *et al.* (2009)'s guidelines. The scope included studies that focused on people's perceptions of and attitudes toward AI, covering various AI use cases adopted at the individual level, enabling us to clearly establish the research objective and set precise inclusion and exclusion criteria for database searches.

### Inclusion criteria

1. Only the academic research focusing on people’s perceptions of or attitudes towards AI and its applications, or the adoption of AI and its various applications by people were included.
2. Only research articles written in English were included.
3. Only research conducted between the years 2015 and 2024 were included.

### Exclusion criteria

1. Articles that focused solely on the computational aspects and techniques of AI applications were excluded.
2. Articles that focused exclusively on AI use cases adopted at a broader level (e.g., by a city or organization) without including any content on those that can be adopted at the individual level were also excluded.
3. Studies that had no content on use cases of AI that could be adopted at the individual level were excluded.

(b) In the screening phase, we manually verified the alignment with the inclusion and exclusion criteria based on the titles and abstracts of the articles. (c) We carried out the eligibility phase on the sample yielded by the previous phase, applying the same criteria checks but on the full text of all samples. Finally, in the (d) inclusion phase, we analysed the remaining included studies. One researcher performed the screening and eligibility assessments, applying the inclusion and exclusion criteria at each phase. The research team then reviewed the results to ensure consistency, address uncertainties, and validate the final selection of studies.

**Figure 1.**
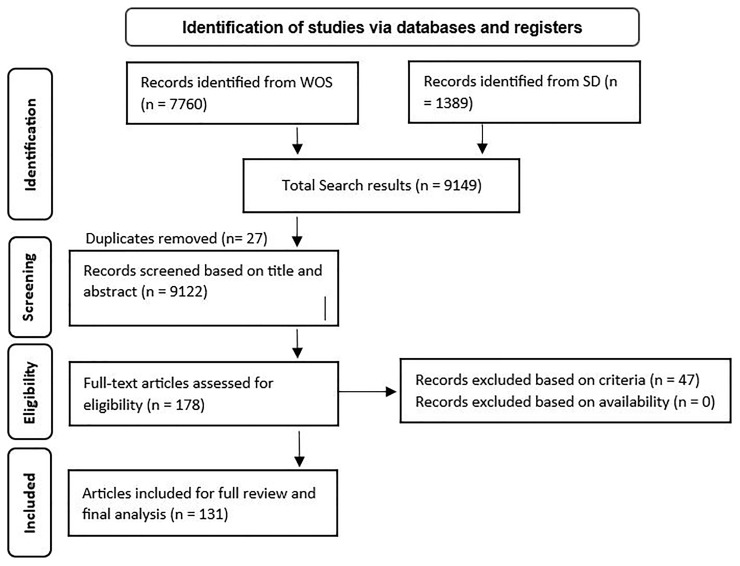
PRISMA flow chart (Adapted from Moher *et al.*, 2009).

As illustrated in Figure 1, the search of the two databases conducted in March 2024 returned 9149 results. We retained 9122 studies after removing the duplicates. Using the inclusion and exclusion criteria, we conducted a filtering of these articles by reading through the title, abstract, and keywords of each one, resulting in 178 articles qualifying for a full-text review. After thoroughly reviewing the full texts and considering only articles that met the inclusion and exclusion criteria, 47 studies were excluded. Ultimately, after excluding also the duplicate results, 131 papers were selected for the final analysis. Refer to the data availability section for the complete list of included articles.

**The analysis:** We analysed the selected articles, and the analyses were recorded and organised in a tabular format (on an Excel worksheet), and the following steps were followed:

- **Indexing the papers:** We documented the title of each paper and assigned it an index number to refer to it (without using the full title, author’s name, etc.) in the tabular analysis.
- **Content Extraction:** We manually reviewed the articles to extract and list the AI use cases that can be adopted at the individual level mentioned in the texts. AI use cases adopted exclusively at a broader level, such as city-level implementations, were not listed. For example, the excluded broader-level AI use cases included city-wide applications such as smart traffic management systems, predictive maintenance in industrial operations, and large-scale climate modelling for environmental monitoring.
- **SDG Correlation:** We linked each AI use case to one or more SDGs based on the authors’ assessment of whether and how it could contribute directly to these goals. Each use case was primarily linked to the SDG for which the connection was most apparent. However, in some cases, a use case was linked to multiple SDGs when the connections were clear. We inferred potential direct linkages to SDGs based on the characteristics of the AI use cases, even if the articles did not explicitly mention any SDG.
- **Impact Scoring:** Subsequently, one of the numerical score values mentioned below was assigned to each linked SDG per AI application based on the paper’s content and narrative:
  - +3, if the authors inferred that the AI use case contributes tremendously positively towards the specific SDG.
  - +2, if the authors inferred that the AI use case contributes moderately positively towards the specific SDG.
  - +1, if authors inferred that the AI use case contributes slightly positively towards the specific SDG.
  - 0, if the authors mentioned the AI use case but did not infer that it contributes positively or negatively towards any SDGs.
  -1 if the authors inferred that the AI use case contributes moderately negatively towards the specific SDG.
  -2 if the authors inferred that the AI use case contributes negatively towards the specific SDG.
  -3 if the authors inferred that the AI use case contributes very negatively towards the specific SDG.

The scoring was conducted relatively, meaning that each score was assigned in comparison to other factors rather than as an absolute measure. In cases where the same AI use cases were mentioned in multiple articles, we assigned impact scores separately for each instance based on the content narrative of the respective paper. The calculated impact score averages provide insights into how AI use cases adopted by people contribute to each SDG.

- **Implementation status:** The authors documented for each article, if the information was present in the body of the article, whether the AI use case has already been implemented in any context or if it is presented as a potential or hypothetical application.
- **Checking whether the narrative on AI’s impact is a hypothesis, based on academic literature or empirical data:** The authors analysed each article to check whether the narratives regarding the impacts of the AI use cases are hypothetical, or derived from existing scientific literature or empirical data.
- **Aggregation of the analyses:** We consolidated the extracted data to identify recurring trends, and patterns regarding AI use cases adopted by people and their association with the Sustainable Development Goals (SDGs). This process involved categorizing AI use cases by their levels of adoption, implementation status, and connections to specific SDGs. Furthermore, to quantify the overall impact of AI use cases on the SDGs, we aggregated the individual impact scores assigned during the analysis phase. For each SDG, the average impact score was calculated by summing the scores of all use cases linked to that SDG and dividing by the total number of valid scores, excluding any "NA" values. The "NA" scores were assigned to denote use cases with a clear connection to an SDG but lacking sufficient information on their impact. Similarly, an overall average impact score was calculated to represent the collective influence of AI use cases on all SDGs. This approach provided a structured assessment of how AI use cases adopted by people contribute to sustainable development goals individually and collectively. We used Excel to extract, organize, and analyse the data throughout this process. One researcher conducted the steps mentioned above. The research team then discussed the results to ensure consistency, resolve uncertainties, and validate the final scoring.

## Findings

### Linking the AI use cases to SDGs

**Figure 2.**
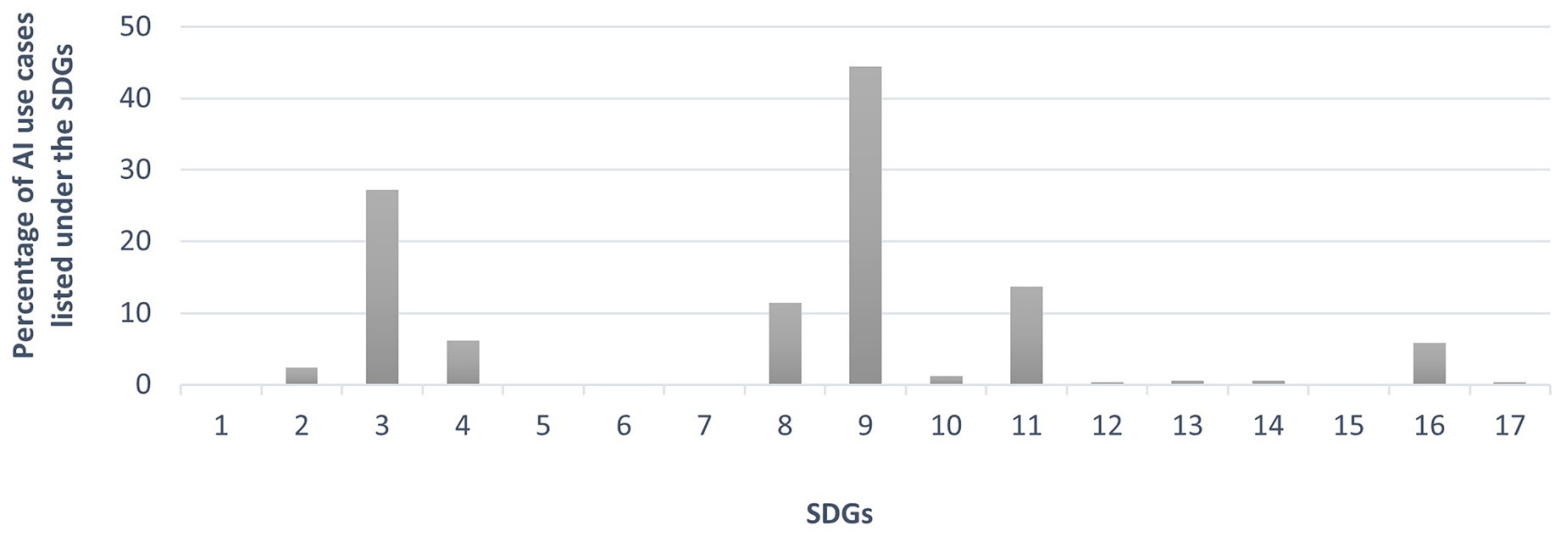
Percentages of AI use cases adopted by people categorised under each SDG (Source: The authors).

We analysed what fraction of the identified AI use cases were linked to each of the Sustainable Development Goals (SDGs). As shown in Figure 2, the AI use cases we identified as being adopted by people are unevenly distributed across the Sustainable Development Goals (SDGs), based on which SDGs the use cases have a direct impact on.

- A significant number of use cases were directly linked to SDG 3 (Good Health and Well-Being: 27.2%), SDG 4 (Quality Education: 6.1%), SDG 8 (Decent Work and Economic Growth: 11.4%), SDG 9 (Industry, Innovation, and Infrastructure: 44.4%), SDG 11 (Sustainable Cities and Communities: 13.7%), and SDG 16 (Peace, Justice, and Strong Institutions: 5.8%).
- Only a limited number of AI use cases had a direct impact on the achievement of SDG 2 (Zero Hunger: 2.3%), SDG 10 (Reduced Inequalities: 1.2%), SDG 12 (Responsible Consumption and Production: 0.3%), SDG 13 (Climate Action: 0.6%), SDG 14 (Life Below Water: 0.6%), and SDG 17 (Partnerships for the Goals: 0.3%).
- None of the AI use cases discussed were directly associated with SDG 1 (No Poverty: 0%), SDG 5 (Gender Equality: 0%), SDG 6 (Clean Water and Sanitation: 0%), SDG 7 (Affordable and Clean Energy: 0%), or SDG 15 (Life on Land: 0%).

### The impact assessment of the AI use cases across the SDGs

Based on the scientific literature we analysed, the overall **average impact of AI use cases adopted by people on the SDGs is 1.02**, indicating that AI use cases adopted by individuals contribute slightly positively to the SDGs. However, we observed that the articles lacked information regarding the impacts of a considerable number of AI use cases (221 out of 342, 64.2%) mentioned within them, in general and in relation to the Sustainable Development Goals (SDGs). These studies focus on other aspects such as acceptance levels or people's perceptions of the AI use cases. For the remaining use cases, we derived impact scores for each AI use case mentioned in the selected articles by analysing their content. Only 4 use cases were framed with hypothetical impacts, offering speculative discussions without evidence or reference. In contrast, 117 use cases provided insights on their impacts supported by references to existing scientific literature.

**Figure 3.**
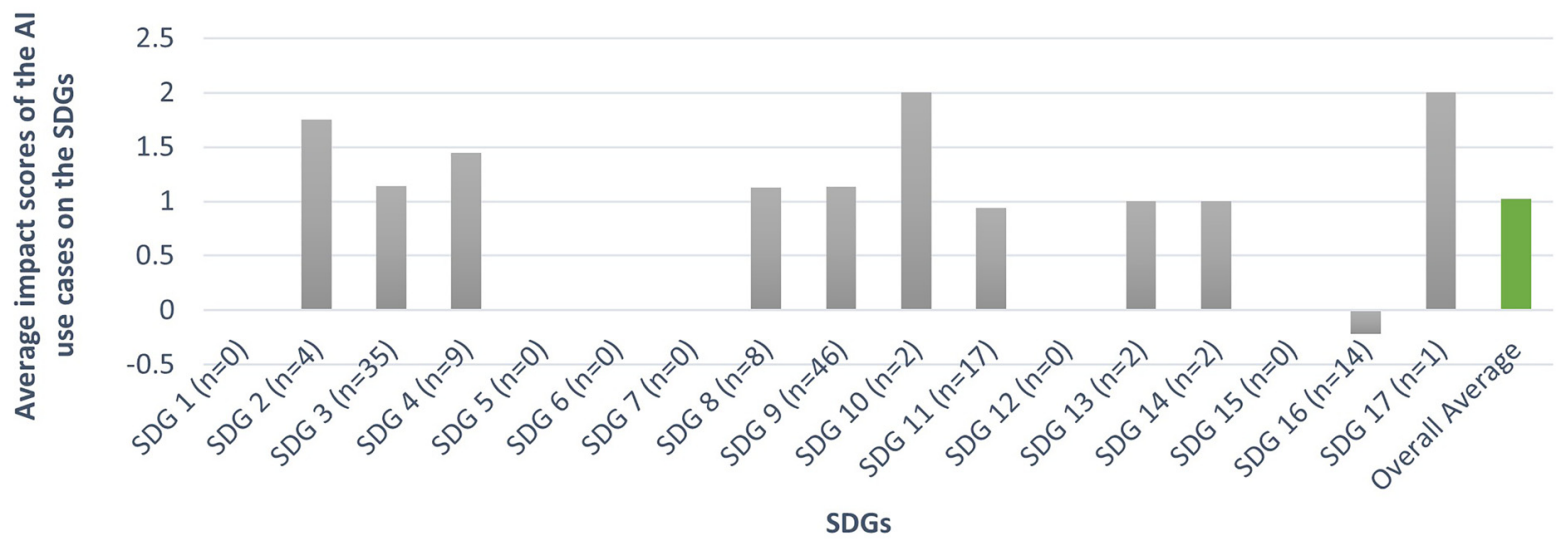
Average impacts of the AI use cases adopted by individuals across the SDGs (n = the number of AI use cases linked to the respective SDG for which we assigned impact scores) (Source: The authors).

As shown in Figure 3, the most notable average impact scores of AI use cases adopted by people across the SDGs were as follows:

- **SDG 2 (Zero Hunger):** Among the 8 use cases, only 4 were assigned impact scores due to a lack of information, resulting in an **average impact score of 1.75**, which showcases AI's potential in advancing agriculture through precision farming and resource optimization.
- **SDG 3 (Good Health and Well-Being):** Of the 93 use cases, only 35 were assigned impact scores, resulting in an **average score of 1.14**, which highlights AI's potential to advance healthcare through improved diagnostics, personalized medicine and fitness plans.
- **SDG 4 (Quality Education):** Out of 21 use cases, 9 were given impact scores, resulting in an **average score of 1.44**, reflecting the positive impact of use cases of AI in facilitating personalized learning and efficient education management.
- **SDG 8 (Decent Work and Economic Growth):** Of the 39 use cases, only 8 were assigned impact scores, with an **average score of 1.13**, suggesting that the adoption of AI by individuals for work boosts economic growth and work efficiency. However, challenges such as job displacement remain a concern.
- **SDG 9 (Industry, Innovation, and Infrastructure):** Of the 152 use cases, 46 were assigned impact scores, with an **average score of 1.13**, indicating AI’s potential in enhancing innovation.
- **SDG 11 (Sustainable Cities and Communities):** Among the 47 use cases, 17 are assessed for impact, resulting in an **average score of 0.94**, showcasing AI’s potential in making cities sustainable through use cases such as navigation support, well-being monitoring, etc.
- **SDG 16 (Peace, Justice, and Strong Institutions):** Out of 20 use cases, 14 report impact scores, with a **negative average score of -0.21**, reflecting concerns about the potential misuse of AI, through use cases such as misinformation, and deceptive chatbots.

However, the variability across SDGs and the negative impact observed for SDG 16 underscore the complexity of AI’s role in achieving sustainable development.

### Implementation status of the AI use cases

The implementation status of the AI use cases reveals that the majority—306 use cases (89.5%)—are already adopted by people in real-world scenarios, showcasing AI's widespread integration into daily life and various sectors. In contrast, 22 use cases (6.4%) lacked information about their implementation status, and 14 use cases (4.1%) were hypothetical, discussed without evidence of real-world application. Examples of hypothetical AI use cases include AI for hairdressing services, and AI algorithms for detecting romance fraud patterns. In some articles, hypothetical scenarios were created to ask questions or collect data on how people might behave in imagined situations involving these AI use cases. For instance, an imaginary scenario with AI in fitness coaching was used to gauge perceptions of feasibility and trust in such technologies. Even though some use cases of AI already exist in real life, they were categorized as hypothetical when the articles analysed did not provide evidence of their real-world implementation or relied on speculative contexts.

## Discussion

By systematically analysing AI use cases adopted by people, identified across 131 research articles, this study demonstrates the widespread integration of AI into people’s lives and its potential to drive sustainable development. However, it also reveals several challenges and gaps that warrant attention.

The analysis confirms AI’s capacity to contribute positively to multiple SDGs when applied thoughtfully and equitably. Notably, use cases related to SDG 3 (Good Health and Well-Being) were the most frequently mentioned, showcasing AI's potential in enhancing diagnostics, personalizing treatments and fitness plans, and providing mental health support. Following this, AI use cases associated with SDG 9 (Industry, Innovation, and Infrastructure) highlighted their positive potential through use cases such as intelligent homes, and content generation. Similarly, AI’s positive impact on SDG 11 (Sustainable Cities and Communities) is evident through applications like navigation systems and disaster response support. Use cases such as personalized education platforms and efficient educational management systems linked to SDG 4 (Quality Education) highlight the positive potential of AI to enhance access to learning and improve overall efficiency in education. The high average impact scores for SDG 2 (Zero Hunger) further demonstrate AI's potential in advancing precision agriculture and improving food security.

Even though our analysis showcases AI's potential to contribute positively to SDG 8 (Decent Work and Economic Growth) by improving cost efficiency and saving time, there are significant challenges associated with its adoption. One major concern is job displacement caused by automation, particularly in sectors reliant on routine tasks, highlighting the need for strategies to support workforce transitions and reskilling. Concerns about fairness and equity also emerge in relation to SDG 10 (Reduced Inequalities), as uneven access to AI technologies and the reinforcement of biases in decision-making systems risk exacerbating social disparities. Furthermore, AI’s potential misuse for privacy violations and fabrication of data, negatively impact SDG 16 (Peace, Justice, and Strong Institutions), and raises ethical questions that call for robust AI governance frameworks. To fully harness AI’s potential for advancing the Sustainable Development Goals (SDGs) and mitigate potential negative impacts, developers and policymakers must ensure that AI use cases are designed and implemented ethically and equitably. Promoting AI literacy is critical, empowering individuals to engage effectively with AI technologies, ensuring equal access, and preventing the widening of social disparities. Additionally, fostering trust in AI through increased explainability and human-centricity is essential for encouraging broader adoption of ethical AI and accelerating progress toward achieving the SDGs.

We compared our main findings with those from existing studies on similar topics. Firstly, studies by Ametepey *et al.* (2024) and Gosselink *et al.* (2024) also concluded that AI's overall contribution to the SDGs is positive. Secondly, consistent with the findings of Vinuesa *et al.* (2020), who analysed use case impacts at a more detailed SDG indicator level ^1^, we observed that AI generally has a positive influence on most SDGs, though it can have negative effects on a smaller fraction of SDGs or their indicators. Finally, researchers such as Lainjo (2024) and Si (2022) emphasize that AI’s potential for sustainable development depends on addressing ethical challenges and mitigating biases—one of our key discussion points as well. This consistency across studies underscores AI’s significant potential for progress, provided it is implemented ethically and responsibly.

A key limitation of this research arises from the inconsistencies in the depth and focus of academic literature on AI’s contributions to sustainability. As a result, we primarily relied on qualitative data extracted from the articles to record the impacts of AI use cases on the SDGs. This over-reliance on qualitative narratives highlights the need for more empirical, data-driven studies that systematically evaluate AI's real-world impacts across diverse contexts and SDGs. Second, our analysis treated SDGs as singular entities, which limits the granularity of insights. A more detailed understanding can be achieved by analysing impacts on the 169 SDG indicators rather than focusing solely on the 17 overarching SDGs. Third, some articles discussed both the positive and negative potential impacts of AI use cases on the same SDG(s). In such cases, we accounted for the positive impacts when it was evident that the negatives could be mitigated through careful development and implementation. Fourth, our analysis is limited to research published in English, which may exclude relevant studies from non-English-speaking contexts. Additionally, the database selection (WOS, ScienceDirect) may have excluded relevant studies from IEEE Xplore or Google Scholar. Last, the single-reviewer screening process introduces potential selection bias.

Future research should prioritize empirical evaluations of AI use cases to provide evidence-based insights into their real-world impacts on the SDGs. A more granular approach is also necessary, analysing impacts on the 169 SDG indicators rather than treating the 17 overarching SDGs as singular entities. Additionally, research should explore the contextual variability of AI's impacts, examining how they differ across regions, populations, and socio-economic settings, with particular attention to marginalised communities. Studies following the suggested format should be conducted separately for AI use cases adopted at the individual level and those implemented at broader levels. Finally, it could be beneficial to record negative and positive impacts separately, as the same AI use case may support or deter progress toward the same SDG(s) or SDG indicators, depending on varying conditions and contexts.

## Ethic and consent

Ethical approval and consent were not required.

## Data Availability

Zenodo: Artificial Intelligence use cases adopted by people and their impact on achieving Sustainable Development Goals. Doi: https://doi.org/10.5281/zenodo.15048897 The dataset ( Palliyil, 2025) supporting this systematic review has been made available publicly on Zenodo and contains the following underlying data: Excel File – Provides a complete list of all studies included in the review, along with all AI use cases adopted at the individual level as mentioned in the studies, the relative magnitude of their impacts on the SDGs, and the source of each impact score—whether hypothetical, derived from empirical research, or adopted from existing literature. Data is available under the terms of the Creative Commons Attribution 4.0 International Zenodo: PRISMA checklist for “Artificial Intelligence use cases adopted by people and their impact on achieving Sustainable Development Goals” Doi: https://doi.org/10.5281/zenodo.15048897 Data is available under the terms of the Creative Commons Attribution 4.0 International Reporting guidelines.
