# Supplementary material for: Artificial Intelligence use cases adopted by people and their impact on achieving sustainable development goals: a systematic review
Source: Open Res Eur. 2026 Feb 10;5:117. Originally published 2025 Apr 28. [Version 2] doi: 10.12688/openreseurope.20023.2 (PMC12949379; doi:10.12688/openreseurope.20023.2)
Supplement: Peer review history

# Reviewer report: Reviewer response for version 2

**DOI:** 10.21956/openreseurope.24974.r69340

**Published:** 2026-02-27

**Authors:** Lóránt Dénes Dávid

**Affiliations:** 1 John von Neumann University, Kecskemét, Hungary

This article has been revised and corrected by the authors; I hereby recommend it for indexing. I could not find any problems in ethical or plagiarism cases.

Are sufficient details of the methods and analysis provided to allow replication by others?

Yes

Are the rationale for, and objectives of, the Systematic Review clearly stated?

Yes

Is the statistical analysis and its interpretation appropriate?

Yes

Are the conclusions drawn adequately supported by the results presented in the review?

Yes

Reviewer Expertise:

tourism, hospitality, geography, earth sciences, economics, environmentalism, regional studies and sciences, sustainable development, circlular economy, bioeconomy, multi-, trans- and interdisciplinary fields

I confirm that I have read this submission and believe that I have an appropriate level of expertise to confirm that it is of an acceptable scientific standard.

# Reviewer report: Reviewer response for version 2

**DOI:** 10.21956/openreseurope.24974.r69577

**Published:** 2026-02-16

**Authors:** Mark A Camilleri

**Affiliations:** 1 University of Malta, Msida, Malta

The authors have already revised their manuscript after they have received the first round of reviewers' evaluations. 

 I believe that their manuscript still has a few weaknesses and shortcomings. I am recommending the following revisions.

 The introduction can include a small section that discusses about the novelty/orginality of this contribution.

 The authors should include more recent references to articles published in 2025 and 2026. They should be drawn from high impact journals. I suggest they include the following ones:

 Al‐Raeei, M. (2025). [Reference 1]

 Costa, A., Crupi, A., Cesaroni, F., & Abbate, T. (2025). [Reference 2].

 Jerez-Jerez, M. J. (2025). [Reference 3]

 Mancuso, I., Petruzzelli, A. M., Panniello, U., & Vaia, G. (2025). [Reference 4]

 Varriale, V., Camilleri, M. A., Cammarano, A., Michelino, F., Müller, J., & Strazzullo, S. (2025). [Reference 5].

 Wang, Q., Zhang, F., & Li, R. (2025).[Reference 6].

 The methodology should better explain the inclusion and exclusion criteria that guided the researchers in their systematic review. The authors need to clarify how they reduced their systematic review of 9122 publications to just 131 items. This would improve the trustworthiness and rigour of this study.

 The findings could have indicated the academic contributions that captured the highest citations in each SDG area.

 The discussion  presents a short discussion considering that the authors examined all SDGs.  The authors need to identify (and elaborate on) the most important themes that emerged from their review.

 This manuscript ought to include a managerial/practical implications section (with at least two paragraphs). The limitations and future research directions should also be presented in another (new) section.

 I look forward to reading a revised version of this manuscript.

Are sufficient details of the methods and analysis provided to allow replication by others?

Partly

Are the rationale for, and objectives of, the Systematic Review clearly stated?

Partly

Is the statistical analysis and its interpretation appropriate?

No

Are the conclusions drawn adequately supported by the results presented in the review?

Yes

Reviewer Expertise:

Strategic Management, Sustainable Development, Circular Economy, Technology Adoption, Digital Transformation, Artificial Intelligence, Education Technology.

I confirm that I have read this submission and believe that I have an appropriate level of expertise to confirm that it is of an acceptable scientific standard, however I have significant reservations, as outlined above.

## References

[1] : The smart future for sustainable development: Artificial intelligence solutions for sustainable urbanization. *Sustainable Development* .2025;33(1) : 10.1002/sd.3131 508-517 10.1002/sd.3131

[2] : Exploring the role of artificial intelligence in addressing sustainable development. A semantic analysis of AI patents. *Technovation* .2025;148: 10.1016/j.technovation.2025.103335 10.1016/j.technovation.2025.103335

[3] : A study of employee attitudes towards AI, its effect on sustainable development goals and non-financial performance in independent hotels. *International Journal of Hospitality Management* .2025;124: 10.1016/j.ijhm.2024.103987 10.1016/j.ijhm.2024.103987

[4] : The bright and dark sides of AI innovation for sustainable development: Understanding the paradoxical tension between value creation and value destruction. *Technovation* .2025;143: 10.1016/j.technovation.2025.103232 10.1016/j.technovation.2025.103232

[5] : Unleashing digital transformation to achieve the sustainable development goals across multiple sectors. *Sustainable Development* .2025;33(1) : 10.1002/sd.3139 565-579 10.1002/sd.3139

[6] : Artificial intelligence and sustainable development during urbanization: Perspectives onAI R&D innovation ,AI infrastructure, andAI market advantage. *Sustainable Development* .2025;33(1) : 10.1002/sd.3150 1136-1156 10.1002/sd.3150

# Reviewer report: Reviewer response for version 1

**DOI:** 10.21956/openreseurope.21658.r61214

**Published:** 2025-10-08

**Authors:** Sina Shaffiee Haghshenas

**Affiliations:** 1 University of Calabria, Rende, Italy

1- The claim of a “significant gap” at the individual level is central—what distinguishes “individual-level adoption” from organizational/city-level beyond examples?

 2- Why restrict to people’s perceptions/attitudes and individual-level adoption but still include SDG 9? Justify inclusion criteria for SDG 9 links at individual level .

 3- The search string ((“people” OR “citizens” OR “residents”) AND (“artificial intelligence” OR “AI”)) is broad; how do you ensure recall/precision and avoid selection bias?

 4- Why exclude IEEE Xplore and Google Scholar given AI application prevalence?

 5- One reviewer conducted screening/eligibility; how is reliability ensured? Use dual-screening on a random subset (e.g., 20%).

 6- How were AI use cases identified and standardized across heterogeneous terminology?

Are sufficient details of the methods and analysis provided to allow replication by others?

Yes

Are the rationale for, and objectives of, the Systematic Review clearly stated?

Yes

Is the statistical analysis and its interpretation appropriate?

Partly

Are the conclusions drawn adequately supported by the results presented in the review?

Partly

Reviewer Expertise:

Transportation Engineering, Road Safety, Smart cities, Sustainable Development, Risk Management, AI, Fuzzy logic, Decision making

I confirm that I have read this submission and believe that I have an appropriate level of expertise to confirm that it is of an acceptable scientific standard, however I have significant reservations, as outlined above.

# Response

**Published:** 2026-02-03

**Authors:** Vijay Gopal Vazhoth Palliyil

**Affiliations:** Department of Civil and Environmental Engineering, TU Darmstadt Institute of Transportation Planning and Traffic Engineering, Darmstadt, Hessen, Germany

We thank the reviewer for the insightful comments and questions, which helped us strengthen the manuscript substantially. Below, we reproduce the reviewer’s comments/questions together with our corresponding responses. Within each response, we indicate the section(s) of the manuscript where we implemented the relevant changes.  

1. The claim of a “significant gap” at the individual level is central—what distinguishes “individual-level adoption” from organizational/city-level beyond examples?

We thank the reviewer for highlighting the need for a clearer conceptual distinction between individual-level and organizational or city-level AI adoption. In this study, *individual-level adoption* refers to AI use cases that individuals voluntarily choose to adopt based on personal preferences and perceived benefits (e.g., personal voice assistants or AI-based learning applications). In contrast, *organizational- or city-level adoption* involves AI systems deployed through institutional or infrastructural decisions typically made by experts or authorities, where individuals are rarely involved in the decision-making process and are often exposed to AI without a meaningful choice or ability to opt out (e.g., AI-enabled traffic management or city-wide surveillance systems). This distinction is central to our research aim, as we examine how **people’s own AI use behaviours and choices**, rather than passive exposure to AI systems, contribute to or hinder progress toward the SDGs. We have clarified this conceptual distinction in the Introduction and Methods sections to make the scope and contribution of the study more explicit.

 2- Why restrict to people’s perceptions/attitudes and individual-level adoption but still include SDG 9? Justify inclusion criteria for SDG 9 links at individual level. We thank the reviewer for this point. Although SDG 9 is often associated with industry and large-scale infrastructure, it also explicitly addresses innovation and technological development, which can be analytically relevant at the individual level when examining AI use cases adopted by people. Accordingly, in this review we linked individual-level AI use cases to SDG 9 when the study narrative emphasised innovation-related or innovation-enabling contributions, such as enabling access to digitally mediated services or supporting technological upgrading and productivity through novel digital tools. More generally, we linked each use case to the SDG(s) that best matched the contribution described in the source article, assigning domain-specific SDGs (e.g., health or education) when the contribution was sectoral and assigning multiple SDGs when more than one clear linkage was described. We have clarified this SDG-assignment logic in the Methods section to make the treatment of SDG 9 at the individual level transparent.

 3- The search string ((“people” OR “citizens” OR “residents”) AND (“artificial intelligence” OR “AI”)) is broad; how do you ensure recall/precision and avoid selection bias? We thank the reviewer for this question. We intentionally used a broad search string to maximize recall across disciplines in a field with heterogeneous terminology. We did not add multiple technique-specific terms (e.g., machine learning, deep learning) because doing so tends to retrieve a higher proportion of studies that focus on computational methods rather than on people’s AI use and experiences. We ensured precision and minimized selection bias through predefined, multi-stage screening at the title/abstract and full-text levels. We included studies if they contained extractable information on at least one AI use case adopted (or adoptable) at the individual level, and we systematically excluded papers focused solely on computational methods or on organizational/city-level deployments without individual-level content. We resolved ambiguous cases through team discussion to ensure consistent application of the criteria. We have updated the Methods section to explicitly describe the rationale for the broad search string and the screening steps used to reduce selection bias.

 4- Why exclude IEEE Xplore and Google Scholar given AI application prevalence? We acknowledge the reviewer’s point regarding IEEE Xplore and Google Scholar. We chose Web of Science and ScienceDirect because they provide broad, multidisciplinary coverage of peer-reviewed literature across AI, social sciences, and sustainability research, and they support a reproducible search process. We recognize that IEEE Xplore and Google Scholar could contain additional relevant studies, particularly conference proceedings and grey literature, and our database selection may therefore have missed some eligible papers. We have added this as a limitation in the manuscript and encourage future work to extend the review by incorporating additional databases.

 5- One reviewer conducted screening/eligibility; how is reliability ensured? Use dual-screening on a random subset (e.g., 20%). We acknowledge that the screening and eligibility assessments were conducted by a single reviewer, which may introduce selection bias. To mitigate this risk, inclusion and exclusion criteria were clearly predefined and applied systematically across the title, abstract, and full-text screening stages, and ambiguous cases were discussed within the research team to reach consensus on study inclusion. We have clarified these procedures in the Methods section and acknowledge that future research could further enhance reliability by applying full or partial dual-screening approaches.

 6- How were AI use cases identified and standardized across heterogeneous terminology? We thank the reviewer for this question. We manually identified and recorded AI use cases adopted (or adoptable) at the individual level from the full texts of the included articles, acknowledging the heterogeneous terminology used across studies. When the same use case appeared in multiple articles, we listed it separately for each study to capture contextual differences and how sustainability impacts were framed. To handle terminological variation, we standardized labels where feasible when studies clearly referred to the same use case; otherwise, we retained the source terminology. We assessed impacts study by study based on each article’s narrative rather than aggregating impact scores across studies. We have clarified this in the Methods section, under the Content Extraction subsection.

# Reviewer report: Reviewer response for version 1

**DOI:** 10.21956/openreseurope.21658.r55578

**Published:** 2025-06-16

**Authors:** Lóránt Dénes Dávid

**Affiliations:** 1 John von Neumann University, Kecskemét, Hungary

I have reviewed the article titled *“Artificial Intelligence Use Cases Adopted by People and Their Impact on Achieving Sustainable Development Goals: A Systematic Review.”*

 This systematic review makes a significant and timely contribution to understanding the role of Artificial Intelligence (AI) in sustainable development from a people-centric perspective. The study stands out by focusing not just on algorithm-centric innovations but on the actual use cases adopted by individuals and their effects - a dimension that is frequently neglected in policy and implementation studies.

 The paper’s main strength lies in its comprehensive and rigorous application of the PRISMA framework to identify, select, and analyze 131 relevant studies. The resulting picture is a well-balanced view: while there are predominantly positive impacts - particularly related to health care, education, innovation, and sustainable cities - there are also important caveats stemming from biases, misinformation, and growing inequalities. This realistic view underscores the necessity for responsible and ethical governance alongside enhanced literacy and policy mechanisms.

 Furthermore, I appreciate your forward-thinking conclusion, which emphasizes future research directions and policy responses to maximize the benefits while mitigating potential drawbacks.

 Suggestions for improvement (with additional citations):

 While the systematic review is exhaustive, I suggest adding more citations to enable readers to appreciate the depth and robustness of the study.

 Recommended articles for citing:

 ( refer to 1,2 and 3)

 Etc.

Are sufficient details of the methods and analysis provided to allow replication by others?

Yes

Are the rationale for, and objectives of, the Systematic Review clearly stated?

Yes

Is the statistical analysis and its interpretation appropriate?

Yes

Are the conclusions drawn adequately supported by the results presented in the review?

Yes

Reviewer Expertise:

tourism, hospitality, geography, earth sciences, economics, environmentalism, regional studies and sciences, sustainable development, circlular economy, bioeconomy, multi-, trans- and interdisciplinary fields

I confirm that I have read this submission and believe that I have an appropriate level of expertise to confirm that it is of an acceptable scientific standard, however I have significant reservations, as outlined above.

## References

[1] https://www.nature.com/articles/s41599-023-01787-8.

[2] https://www.sciencedirect.com/science/article/abs/pii/S0019850123002250.

[3] https://www.frontiersin.org/journals/forests-and-global-change/articles/10.3389/ffgc.2024.1409667/full.

# Response

**Published:** 2026-02-03

**Authors:** Vijay Gopal Vazhoth Palliyil

**Affiliations:** Department of Civil and Environmental Engineering, TU Darmstadt Institute of Transportation Planning and Traffic Engineering, Darmstadt, Hessen, Germany

We thank the reviewer for the positive assessment of our work and for the helpful suggested references. In response, we have incorporated the relevant recommended literature where it aligns directly with the focus of our review, to strengthen both (i) the contextual grounding of our discussion, particularly around the responsible and ethical development and use of AI, and (ii) the methodological positioning of our SDG-linkage procedure by referencing a prior PRISMA-based review that similarly map extracted study content to the SDGs.
